# Eliminating Radiation Resistance of Non-Small Cell Lung Cancer by Dihydroartemisinin Through Abrogating Immunity Escaping and Promoting Radiation Sensitivity by Inhibiting PD-L1 Expression

**DOI:** 10.3389/fonc.2020.595466

**Published:** 2020-10-28

**Authors:** Hai Zhang, Fei Zhou, Yingying Wang, Huikang Xie, Shilan Luo, Lu Meng, Bin Su, Ying Ye, Kailiang Wu, Yaping Xu, Xiaomei Gong

**Affiliations:** ^1^Department of Pharmacy, Shanghai First Maternity and Infant Hospital, Tongji University School of Medicine, Shanghai, China; ^2^Department of Oncology, Shanghai Pulmonary Hospital, Tongji University School of Medicine, Shanghai, China; ^3^Department of Radiation Oncology, Shanghai Pulmonary Hospital, Tongji University School of Medicine, Shanghai, China; ^4^Department of Pathology, Shanghai Pulmonary Hospital, Tongji University School of Medicine, Shanghai, China; ^5^Department of Radiation Oncology, Fudan University Shanghai Cancer Center, Shanghai, China

**Keywords:** radiation resistance, synergistic action, dihydroartemisinin, radiotherapy, non-small cell lung cancer, programmed death ligand 1

## Abstract

Radiation resistance is linked to immune escaping and radiation sensitivity. In this study, we found that the PD-L1 expressions of non-killed tumor cells in NSCLC were enhanced after radiotherapy, and dihydroartemisinin (DHA) could synergistically enhance the antitumor effect of radiotherapy in NSCLC. A total of 48 NSCLC patients with sufficient tumor tissues for further analyses were enrolled. The PD-L1 expressions of NSCLC were evaluated by immunohistochemistry. Cell apoptosis was measured by flow cytometry, and the relationship between the PD-L1 expression and radiation resistance was investigated in patient specimens, xenograft model, and cell lines. First, the results indicate that the PD-L1 expression of NSCLC was positively related with the radiation resistance. Second, we found that DHA could eliminate the radiation resistance and synergistically enhance the antitumor effect of radiotherapy in the NSCLC cells lines and xenograft model. Finally, mechanistically, DHA could inhibit the PD-L1 expression to avoid immune escaping by inhibiting TGF-β, PI3K/Akt, and STAT3 signaling pathways. In addition, DHA could activate TRIM21 and regulate the EMT-related proteins by inhibiting the PD-L1 so as to enhance the radiation sensitivity and eliminate radiation resistance to NSCLC. Collectively, this study established a basis for the rational design of integrated radiotherapy and DHA for the treatment of NSCLC.

## Introduction

Lung cancer is the main cause of cancer-related deaths in China, and non-small cell lung cancer (NSCLC) accounts for approximately 80% of all lung cancers (1). About 70% of NSCLC patients need radiotherapy during treatment, which is the most import nonsurgical treatment strategy in the multidisciplinary management of NSCLC patients (2). However, radiation resistance is still a major clinical problem, leading to poor prognosis of cancer patients (3–5).

NSCLC is a highly immunosuppressed malignant tumor, and numerous mechanisms have been reported to evade the antitumor immune response, including immunomodulatory cytokine release, inhibition of T cell activation, and other defects (5, 6). Radiotherapy kills cancer cells *via* the ionizing radiation of X, γ, or high-energy electron beams by destroying the DNA of tumor cells so it can have targeted therapeutic effects without damaging normal cells (7). However, not all NSCLC patients have a good response to radiotherapy. Previous studies related to radiotherapy mainly focus on the tumor cell itself, ignoring the immune system in the tumor microenvironment. Recently, increasing evidence has identified a close link between radiation resistance and immunity evasion because the radiotherapy effect and radiation sensitivity vary from person to person (4, 8–13). It has also been reported that some specific signaling pathways contribute to radiation resistance, such as DNA damage repair, cell adhesion, immune checkpoint block, and inflammatory response (4, 7, 14–18).

The programmed cell death ligand 1 (PD-L1) are important immune checkpoint molecules restricting the antitumor immune response and inducing immune escape (9, 12, 13). Our previous studies also show that NSCLC patients with high PD-L1 expressions produce radiotherapy resistance, and their radiotherapy effect is worse than those with negative PD-L1 expressions (19, 20). Meanwhile, it has also been proven that the radiotherapy effect can be enhanced with PD-L1 antibodies in the NSCLC cells and mice (19, 21).

PD-1/PD-L1 antibodies have been widely used in the treatment of NSCLC, but they are often accompanied with unpredicted side effects, and many patients cannot derive survival benefits with high medical expenses (4, 11, 22). Therefore, it is promising to find a small molecule compound inhibiting the PD-L1 expression that could be used in combination with radiotherapy for the treatment of NSCLC. Artemisinin is a famous sesquiterpene lactone endoperoxide in *Artemisia annua* L for its derivatives being effective FDA-approved and WHO-recommended for malaria treatment pillars (23–25). Dihydroartemisinin (DHA) is an active metabolite of many artemisinin compounds in *Artemisia annua* L and is 5 times more potent than artemisinin against malaria (26, 27). Remarkably, DHA demonstrates good inhibitory effects on numerous tumors, including hepatocellular carcinoma, pancreatic cancer, and lung cancer, et al. (28–33). More interestingly, DHA can enhance radiation sensitivity to glioblastoma, cervical cancer, and lung cancer, et al. by regulating inflammatory signaling pathways, such as nuclear factor kappa-B (NF-Ƙb) and extracellular signal regulated protein kinase 1/2 (ERK1/2) activity (20, 34–36). Therefore, we speculated that integrated radiotherapy and DHA may have better therapeutic effects.

In the present study, we explored the relationship between the PD-L1 expression and radiation resistance in NSCLC and then evaluated the synergistic action of radiotherapy and DHA and, finally, elucidated the potential synergistic mechanisms in eliminating radiation resistance and enhancing the radiotherapy effect in NSCLC cells and xenograft mice.

## Methods

### Patients and Tissue Collection

A total of 57 patients with recurrent NSCLC after surgical resection, who received concurrent chemoradiotherapy from January 2014 to December 2019 at Shanghai Pulmonary Hospital (Shanghai, China) were identified in the present study. All patients had adequate information for response assessment after delivery of conventionally fractionated radiotherapy. Tumor tissues were fixed in 10% neutral-buffered formalin and stored as paraffin-embedded archival (FFPE) samples. All tissues were reviewed by experienced pathologists for confirmation of histological type and a tumor content higher than 30%. PD-L1 immunohistochemistry testing was performed on FFPE samples. The tumor response was assessed according to the Response Evaluation Criteria in Solid Tumor (RECIST, version 1.1). This study was approved by the Ethics Committee of Shanghai Pulmonary Hospital, Tongji University School of Medicine (K16-202), and written informed consent was obtained from each participant before any study-related procedures.

### Cell Culture and Cell Transfection

The A549, PC9, and Lewis lung cancer cells (LLC) were obtained from ATCC (Manassas, VA, USA). The A549/X and PC9/X cell lines were induced and obtained in our research laboratory as previously described (15, 19). All cell lines were cultured in Dulbecco’s modified Eagle medium (DMEM) (Hyclone, Longan, UT, USA) containing 10% fetal bovine serum (FBS) (Life Technologies, Grand Island, NY) at 37°C with 5% CO_2_. All cell lines for research were cultured less than 20 generations and were routinely screened to confirm the absence of Mycoplasma contamination.

Cell transfection experiments with PD-L1 mimics and siRNAs (RiboBio, Guangzhou, China) and their corresponding controls were carried out with 60%–70% confluent cells grown in 6-well plates by using Lipofectamine 2000 (Invitrogen, Carlsbad, CA, USA) according to the manufacturer’s instructions as previously described (15, 19). The target sequence for si-PD-L1-RNA was as follows: sense strand, 50-CAUAGUAGCUACAGACAGA dTdT-30, antisense strand, and 30-dTdT GUAUCAUCGAUGUCUGUCU-50. Forty-eight hours after transfection, the cells were harvested for flow cytometry or western blot analyses.

### Cell Proliferation and Apoptosis Assays

Cells were seeded overnight at a density of 1×10^5^ in 96-well plates in DMEM containing 10% FBS, and an MTT assay was used to determine the proliferation of different cells exposed to DHA (Sigma, USA). After growing for 48 h, the cells were incubated for 4 h at 37°C with 200 μl MTT solution and measured at 490 nm by using a microplate reader.

For apoptosis assay, the cells were harvested, washed twice with pre-cold PBS, centrifuged at 1000 rpm for 10 min, and resuspended in chilled PBS after growing for 48 h in DMEM containing 10% FBS. Then, the cells were stained with fluorescein-conjugated annexin V and propidium iodide (BD Biosciences) for 15 minutes in the dark at room temperature according to the manufacturer’s instructions for the apoptosis staining, and the percentages of apoptosis cells were analyzed by a BD FACSCalibur flow cytometer (BD Biosciences, San Jose, CA). The experimental groups are representative of at least three independent experiments.

### Western Blot Analyses

Cells were lysed using RIPA protein extraction reagent (Beyotime, Beijing, China) supplemented with phenylmethanesulfonyl fluoride (PMSF) (Riche, CA, USA). Approximately 50 µg of protein extracts were separated by 10% sodium dodecyl sulfate polyacrylamide gel electrophoresis (SDS-PAGE), transferred onto nitrocellulose membranes (Sigma), and incubated with specific antibodies. An enhanced chemiluminescent (ECL) chromogenic substrate was used to visualize the bands. The blots were developed with a chemiluminescence system, and GAPDH was used as a control. All the rabbit monoclonal antibodies were purchased from Cell Signaling Technology, including PD-L1 (1:5000), Bim, Bcl-2, cleaved caspases-3, cleaved caspases-8 (1:5000), TGF-β, TRIM21, NF-ƘB, AKT Phospho-AKT (Ser473) (1:2500), AKT (1:2500), Phospho-STAT3 (Tyr705) (1:3000), STAT3 (1:3000), E-cadherin, N-cadherin, Snail, and Vimentin (1:1000). Experimental groups are representative of at least 3 independent experiments.

### Xenograft Animal Study

The 5-week-old female specific pathogen-free (SPF) C57/BL6 mice were used for animal experiments. The animal studies were approved by the Institutional Animal Care and Use Committee of Tongji University School of Medicine and were performed according to institutional guidelines. The LLC cells were injected into the right outer thighs of the mice. Radiotherapy was commenced once a day for 5 days at 2 Gy of the fractionated radiotherapy on day 1, and DHA was intragastric administered once a day for 12 days at a dose of 50 mg/kg as Gong and Zhou et al. previously described (19, 37).

Tumor sizes were assessed every 2 days by a digital caliper. The tumor volumes were determined by measuring their length (l) and width (w) and calculating the volume (V) as follows: V=lw^2^/2. After 12 days of treatment with DHA, the mice were killed and paraffin-embedded tissues were prepared for immunohistochemistry staining. Experimental groups containing at least 6 mice/group are representative of at least 3 independent experiments.

### Immunohistochemistry (IHC)

The 4-µm-thick formalin-fixed paraffin-embedded (FFPE) xenograft NSCLC tumors were dewaxed in xylene, hydrated in graded alcohols, and washed with PBS. After blocking endogenous peroxidase activity with 3% H_2_O_2_ aqueous solution for 10 minutes, the sections were incubated with primary antibodies overnight. After washing with PBS, they were then incubated with general-type IgG-HRP Polymer (K5007, Dako, USA) for 10 min, followed by 3, 3’-diaminobenzidine (DAB) for about 2 to 5 min. Finally, the sections were restained with hematoxylin for 1 min and then dehydrated in graded alcohols, cleared in xylene, and covered with coverslips. We adopted the staining-intensity-distribution (SID) score as previously described by Ye et al. (18). The intensity of positive tumor cells was categorized into three grades according to staining intensities compared with those of internal controls: -, negative staining; + (weak), lighter than skeletal muscle; ++ (moderate), equal to skeletal muscle; and +++ (strong), more intense than skeletal muscle. The distribution of positive tumor cells was graded as -, no stained cells; +, <25% stained cells; ++, 25%–50% stained cells; and +++, >50% stained cells. We used rabbit polyclonal to PD-L1 (1:100), CD8 (1:100), CD4 (1:500), Foxp3 (1:500), LyGr (1:1000) and CD11b (1:1000) as primary antibodies (Novus Biologicals, USA). Experimental groups are representative of at least 3 independent experiments.

### Statistical Analysis

Quantitative values were presented as mean ± standard deviation (SD) or standard error of mean (SEM) of 3 independent assays. The independent sample *t* test was used to compare the mean values, and *χ^2^* test and Fisher’s exact test were used to compare categorical value. The two-sided *P*<0.05 was consider to be statistically significant. Statistical analysis was performed using SPSS software package (version 17.0, Chicago, SPSS Inc.).

## Results

### High PD-L1 Expressions Were Associated With Radiation Resistance and Poor Prognosis in NSCLC Patients Treated With Radiotherapy

**Figure 1:**
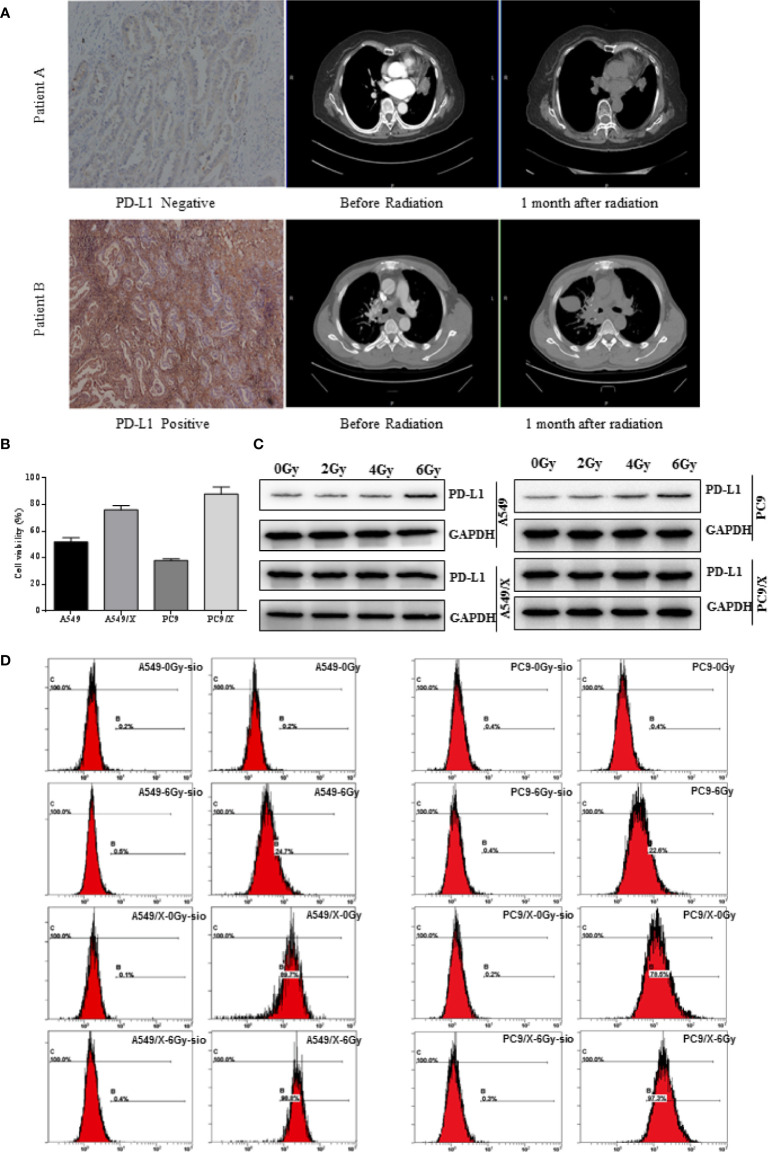
The PD-L1 expression of NSCLC was positively related with the radiation resistance, and radiotherapy enhances PD-L1 expression of NSCLC cells. **(A)** The two representative patients with negative and positive PD-L1 expression achieved different responses to radiotherapy. **(B)** Growth inhibition of the A549, A459/X, PC9, and PC9/X cell lines after delivery of 2 Gy of radiation per fraction by MTT assay. **(C)** The expression of PD-L1 in A549, A459/X, PC9, and PC9/X cell lines after conventionally fractionated radiotherapy (0, 2, 4, and 6 Gy in 2 Gy per fraction) lines by Western blot. **(D)** The expression of PD-L1 in A549, A459/X, PC9, and PC9/X cell after 6 Gy was delivered in three fractions *in vitro* by flow cytometry.

The relationship between PD-L1 expression and tumor regression was evaluated in 57 NSCLC patients who received radiotherapy. Notably, it was observed that the patients with 18 positive expressions of PD-L1 achieved a good response to radiotherapy, and the 39 patients with PD-L1 positive tumors had a relatively lower response to radiotherapy. The ORRs (100% vs. 56.41% [*p < 0.001*]) were significantly higher in patients with negative PD-L1 expression (IHC 0) than those with positive PD-L1 expression (IHC 1, 2, and 3) (**Table 1**), suggesting that the expression of PD-L1 was positively related with the radiation resistance in NSCLC. Patients with negative PD-L1 expression showed a good response to radiotherapy, and those with high PD-L1 expression showed minimal or no response to radiotherapy (**Figure 1A**). These results demonstrate that high PD-L1 expressions may promote radiation resistance and was associated with poor prognosis after radiotherapy in NSCLC patients. Although patients with negative PD-L1 expression showed a better radiotherapy effect in the first radiotherapy, its radiotherapy effect gradually decreased. It was deduced that radiotherapy not only kills some of NSCLC cells but also increases the expression of PD-L1 in non-killed NSCLC cells, and therefore, the expression of PD-L1 in NSCLC patients with negative PD-L1 expression are enhanced after multiple radiotherapy.

**Table 1:** The Characteristics of Efficacy, Radiotherapy Response, and PD-L1 Expression in Patients with NSCLC.

Characteristic	Overall	PD-L1 expression (n=57)				P value
		IHC 0 (n=18)	IHC 1 (n=21)	IHC 2 (n=16)	IHC 3 (n=2)	
Age, year						0.018
≥65	25	10	9	6	0	
<65	32	8	12	10	2	
Sex						1.003
Male	41	16	13	11	1	
Female	16	2	8	5	1	
T stage						0.021
T1	19	9	7	3	0	
T2	27	6	8	12	1	
T3	7	3	3	1	0	
T4	4	0	3	0	1	
N stage						
N0	19	7	9	5	0	0.051
N1	17	6	5	6	0	
N2	11	3	4	3	1	
N3	8	2	3	2	1	
TNM stage						0.049
I	23	8	8	7	0	
II	18	5	6	6	1	
III	16	5	7	3	1	
Efficacy						
PR	40	18	16	6	0	
SD	14	0	5	9	0	
PD	3	0	0	1	2	
ORR	71.4%	100%	76.19%	37.5%	0	0.001

Negative PD-L1 expression (IHC 0); Positive PD-L1 expression (IHC 1, 2, and 3); PD-L1, programmed death 1 ligand; IHC, immunohistochemistry; PR, partial response; PD, progressive disease; ORR, objective response rate.

### The Expression of PD-L1 Was Enhanced After Radiotherapy and Positively Related With the Radiation Resistance in NSCLC Cell Lines

To further investigate the role of PD-L1 involved in radiation resistance, the A549 and PC9 radiation resistance cell lines were induced and named A549/X and PC/R as we described previously. Given delivery of 2 Gy radiation, the cell viability of A549/X (76%) and PC9/X (88%) were higher than A549 (52%) and PC9 (38%), suggesting that the A549/X and PC9/X cells had already been resistant to radiation (**Figure 1B**). Western blot analyses showed that PD-L1 expression was significantly upregulated in radiation-resistant NSCLC cell lines (A549/X and PC9/X cells, **Figure 1C**) compared to normal NSCLC cells lines (A549 and PC9 cells, **Figure 1C**). After conventionally fractionated radiation at doses of 0, 2, 4, and 6 Gy (2 Gy per fraction per day for 3 consecutive days), the expression of PD-L1 in survival A549 and PC9 cells sorted by flow cytometry significantly increased, but that in radioresistant A549/X and PC9/X cells was in a high expression state with no significant change. (**Figure 1D**). These results suggest that conventionally fractionated radiation upregulates the PD-L1 expressions of A549 and PC9 cells and elevated the PD-L1 expression was associated with radiation resistance of NSCLC cells.

### DHA Eliminates Radiation Resistance of NSCLC Cell Lines by Inhibiting the PD-L1 Expression

**Figure 2:**
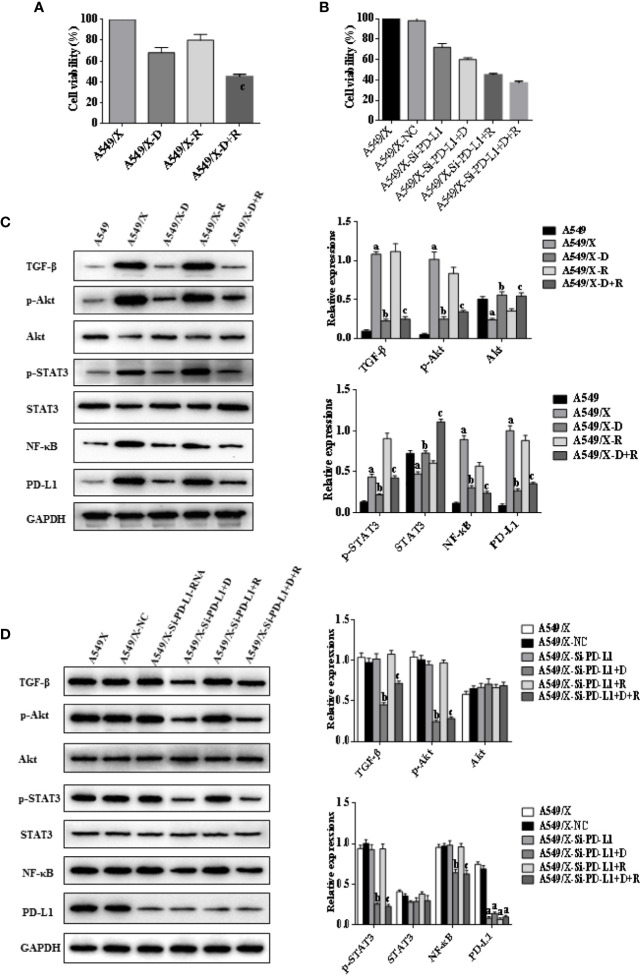
Effects of radiotherapy and DHA on PD-L1, TGF-β, PI3K/Akt, and STAT3 signaling pathways in A549 and A549/X cells. **(A)** The MTT assay results of A549 and A549/X cell lines treated with vehicle, D, R, D+R. **(B)** The MTT assay results of A549/X-NC and A549/X-Si-PD-L1-RNA cell lines treated with vehicle, D, R, D+R. **(C)** The expressions of TGF-β, Akt, p-Akt, STAT3, p-STAT3, NF-κB, and PD-L1 in A549 and A549/X cell lines treated with vehicle, D, R, D+R by western blot. The values are expressed as means ± SEM. ^a^*p* < 0.05 vs. the A549 group, ^b^*p*<0.05 vs. the A549/X group, ^c^*p* < 0.05 vs. the A549/X-R group; **(D)** The expressions of TGF-β, Akt, p-Akt, STAT3, p-STAT3, NF-κB, and PD-L1 in A549/X transfected with NC or PD-L1 siNRA while treated with vehicle, D, R, D+R by western blot. The values were expressed as means ± SEM. ^a^*p* < 0.05 vs. the A549/X-NC group, ^b^*p* < 0.05 vs. the A549/X-Si-PD-L1-RNA group, ^c^*p* < 0.05 vs. the A549/X-Si-PD-L1-RNA+R group.

**Figure 3:**
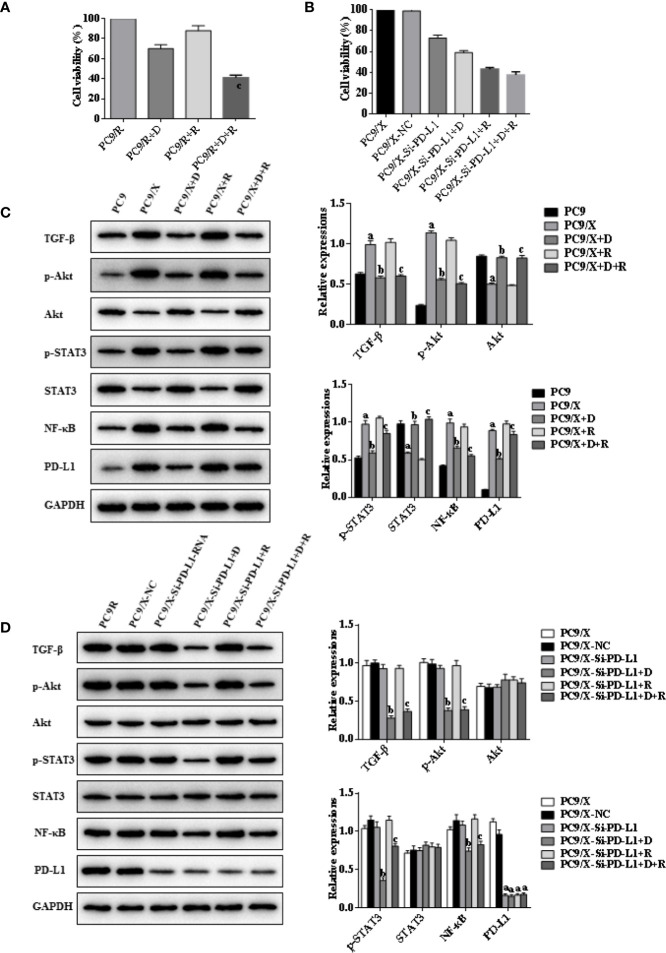
Effects of radiotherapy and DHA on PD-L1, TGF-β, PI3K/Akt, and STAT3 signaling pathways in PC9 and PC9/X cells. **(A)** The MTT assay results of PC9 and PC9/X cell lines treated with vehicle, D, R, D+R. **(B)** The MTT assay results of PC9/X-NC and PC9/X-Si-PD-L1-RNA cell lines treated with vehicle, D, R, D+R. **(C)** The expressions of TGF-β, Akt, p-Akt, STAT3, p-STAT3, NF-κB, and PD-L1 in PC9 and PC9/X cell lines treated with vehicle, D, R, D+R by western blot. The values are expressed as means ± SEM. ^a^*p* < 0.05 vs. the PC9 group, ^b^*p* < 0.05 vs. the PC9/X group, ^c^*p* < 0.05 vs. the PC9/X-R group; **(D)** The expressions of TGF-β, Akt, p-Akt, STAT3, p-STAT3, NF-κB, and PD-L1 in PC9/X transfected with NC or PD-L1 siNRA while treated with vehicle, D, R, D+R by western blot. The values are expressed as means ± SEM. ^a^*p* < 0.05 vs. the PC9/X-NC group, ^b^*p* < 0.05 vs. the PC9/X-Si-PD-L1-RNA group, ^c^*p* < 0.05 vs. the PC9/X-Si-PD-L1-RNA+R group.

**Figure 4:**
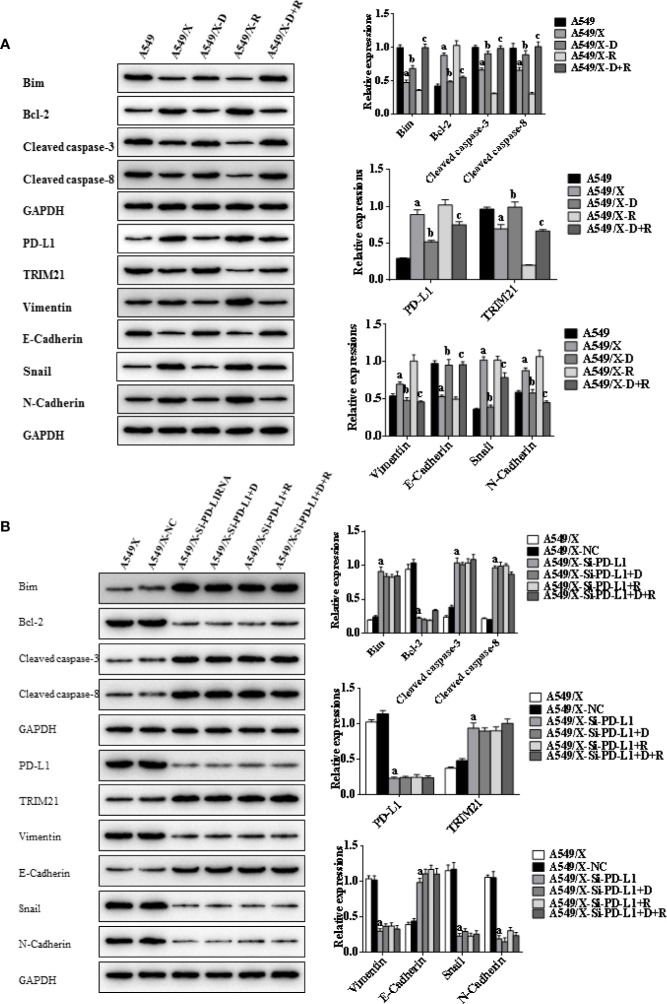
Effects of radiotherapy and DHA on apoptosis-related signaling pathways in A549 and A549/X cells. **(A)** The expressions of TRIM21, apoptosis-related proteins Bcl-2, cleaved caspases-3, cleaved caspases-8, Bim, and EMT-related proteins Vimentin, E-Cadherin, Snail, and N-Cadherin in A549 and A549/X cell lines treated with vehicle, D, R, D+R by western blot. The values are expressed as means ± SEM. ^a^*p* < 0.05 vs. the A549 group, ^b^*p* < 0.05 vs. the A549/X group, ^c^*p* < 0.05 vs. the A549/X-R group. **(B)** The expressions of TRIM21, apoptosis-related proteins Bcl-2, cleaved caspases-3, cleaved caspases-8, Bim, and EMT-related proteins Vimentin, E-Cadherin, Snail, and N-Cadherin in A549/X, A549/X-NC, and A549/X-Si-PD-L1 treated with vehicle, D, R, D+R by western blot. The values are expressed as means ± SEM. ^a^*p* < 0.05 vs. the A549/X-NC group, ^b^*p* < 0.05 vs. the A549/X-Si-PD-L1 group, ^c^*p* < 0.05 vs. the A549/X-Si-PD-L1+R group.

**Figure 5:**
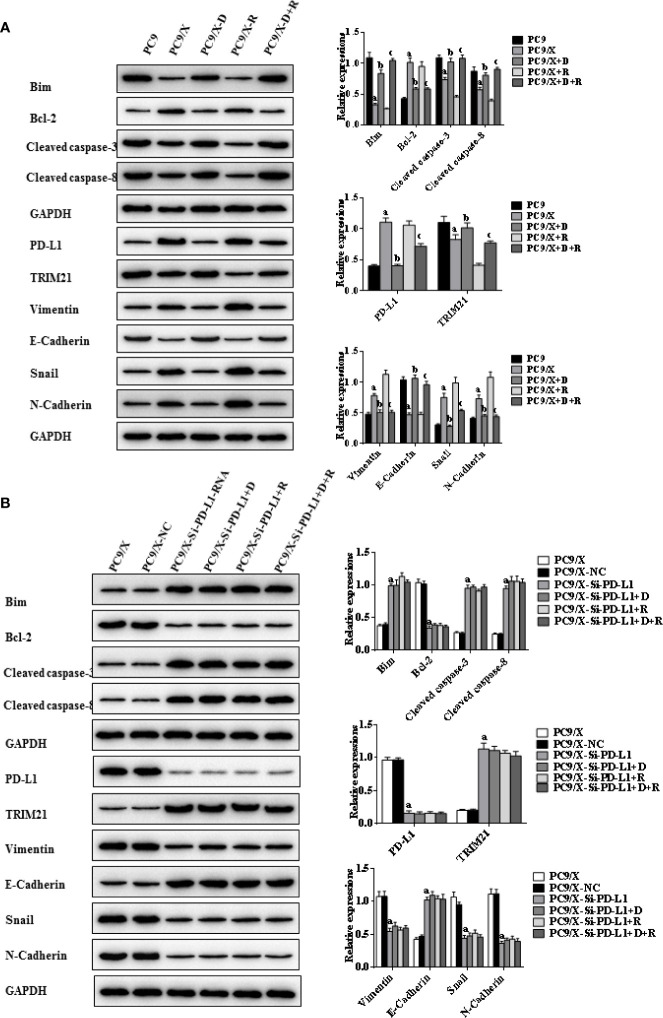
Effects of radiotherapy and DHA on apoptosis-related signaling pathways in PC9 and PC9/X cells. **(A)** The expressions of TRIM21, apoptosis-related proteins Bcl-2, cleaved caspases-3, cleaved caspases-8, Bim, and EMT-related proteins Vimentin, E-Cadherin, Snail, and N-Cadherin in PC9 and PC9/X cell lines treated with vehicle, D, R, D+R by western blot. The values are expressed as means ± SEM. ^a^*p*<0.05 vs. the PC9 group, ^b^*p*<0.05 vs. the PC9/X group, ^c^*p*<0.05 vs. the PC9/X-R group. **(B)** The expressions of TRIM21, apoptosis-related proteins Bcl-2, cleaved caspases-3, cleaved caspases-8, Bim, and EMT-related proteins Vimentin, E-Cadherin, Snail, and N-Cadherin in PC9/X, PC9/X-NC, and PC9/X-Si-PD-L1 treated with vehicle, D, R, D+R by western blot. The values were expressed as means ± SEM. ^a^*p*<0.05 vs. the PC9/X-NC group, ^b^*p*<0.05 vs. the PC9/X-Si-PD-L1 group, ^c^*p*<0.05 vs. the PC9/X-Si-PD-L1+R group.

Whether DHA could eliminate radiation resistance of NSCLC cells was further analyzed. An MTT cell viability assay showed that cell viability of radiation-treated A549/X and PC9/X cells was significantly decreased compared to A549 and PC9 cells after treatment with DHA (**Figures 2A**, **3A**). When the PD-L1 was knocked down, it was found that the cell viability of radiation-treated A549/X-Si-PD-L1-RNA and PC9/X-Si-PD-L1-RNA cells was also decreased significantly, whose effects were similar as DHA (**Figures 2B**, **3B**). The apoptosis rate of A549/X and PC9/X cells was not significantly changed after a delivery of 6 Gy radiation (1.0% vs. 1.3%), suggesting that A549/X and PC9/X cells were resistant to radiation. It was noteworthy that the apoptosis rate of radiation-treated A549/X and PC9/X cells significantly increased after treatment with DHA (71.5% and 68.2%), suggesting that DHA could alleviate radiation resistance and promote apoptosis (**Figures 4A**, **5A**). When PD-L1 was knocked down, we found that the apoptosis rate of radiation-treated A549/X-Si-PD-L1-RNA and PC9/X-Si-PD-L1-RNA cells was also significantly increased, and the effects were similar to DHA. PD-L1 knockdown can achieve a similar effect as DHA on the cell viability and apoptosis rate of radiation-treated A549/X and PC9/X cells. Western blot analyses indicated that the PD-L1 expression in A549/X and PC9/X cells was significantly decreased after treatment with DHA (**Figures 2D**, **3D**). Taken together, these results indicate that DHA eliminates radiation resistance of NSCLC cell lines by inhibiting PD-L1 expression.

### DHA Downregulates the PD-L1 Expression to Evade Immunity Escaping Through Inhibition of TGF-β, PI3K/AKT, and STAT3 Signaling Pathways

The western blot results indicated that the expressions of TGF-β, PD-L1, p-AKT, and p-STAT3 were upregulated after delivery of radiation in A549/X and PC9/X cells, and those were downregulated after treatment of DHA (**Figures 2C**, **3C**). More notably, the expression of TGF-β, p-AKT, p-STAT3, and PD-L1 were all reversed after integrated DHA and radiation treatment significantly more than those of radiation alone, suggesting the synergistic antitumor effect might be driven by the inhibition of TGF-β, p-AKT, p-STAT3, and PD-L1 (**Figures 2C**, **3C**). Meanwhile, we also noticed that the expression of p-AKT, p-STAT3, and NF-ƘB was not changed after transfection with si-PD-L1-RNA in A549/X and PC9/X cells but decreased after treatment of DHA (**Figures 2D**, **3D**), suggesting that PI3K/AKT and STAT3 are the upstream signaling pathways of PD-L1, and DHA downregulates the PD-L1 expression to eliminate radiation resistance by inhibiting the upstream PI3K/AKT, STAT3, and NF-ƘB signaling pathways.

### DHA Promotes Radiation Sensitivity to Facilitate Cell Apotosis by Activating PD-L1-Independent Downstream Signaling Pathways Trim21 and EMT-Related Proteins

Indicators of cell apoptosis, including BIM, caspases-8, caspases-3, and Bcl-2, were analyzed by western blot. The western blot results showed that the expression of pro-apoptosis protein BIM was significantly upregulated, the cleavage of caspases-8 and caspases-3 was promoted, and the expression of anti-apoptosis protein Bcl-2 was downregulated after integrating DHA and radiation treatment (**Figures 4A**, **5A**). In addition, E-cadherin, vimentin, Snail, and N-cadherin are all involved in the EMT process, whose expressions can reflect the rate of cell apoptosis. The expression of E-cadherin was significantly increased in A549/X and PC9/X cells treated with radiotherapy and DHA, and the expression of vimentin, Snail, and N-cadherin were decreased (**Figures 4A**, **5A**), implicating that DHA may enhance radiation sensitivity *via* inhibiting EMT process. As shown in **Figures 4A**, **5A**, the expression of TRIM21 protein was increased after treatment with radiotherapy and DHA.

When the PD-L1 was knocked down, the apoptosis rate of radiation-treated A549/X-Si-PD-L1-RNA and PC9/X-Si-PD-L1-RNA cells were also significantly increased. Western blot results showed that the pro-apoptosis protein BIM was significantly upregulated, anti-apoptosis protein Bcl-2 was downregulated, TRIM21 protein was increased, and the cleavage of caspases-8 and caspases-3 was promoted, and these signaling pathways were not changed significantly after treatment of radiotherapy and DHA (**Figures 4B**, **5B**). These results above indicated that TRIM21 and EMT-related proteins were PD-L1-dependent downstream signaling pathways. Taken together, DHA could promote radiation sensitivity to facilitate cell apoptosis by activating PD-L1-dependent downstream signaling pathways TRIM21 and EMT-related proteins.

### Synergistic Actions of Integrated Radiotherapy and DHA for the Treatment of Anticancer of NSCLC In Xenograft Mice

**Figure 6:**
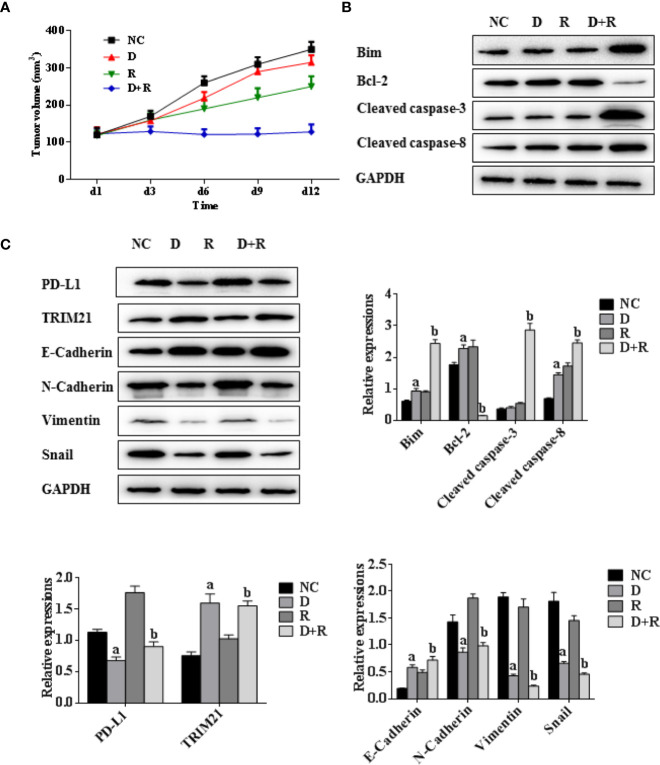
Synergistic actions of radiotherapy and DHA on NSCLC in xenograft model mice. **(A)** Tumor volumes of xenograft model mice treated with vehicle, D, R, D+R *in vivo* after 12 days. **(B)** The expressions of apoptosis-related proteins Bcl-2, cleaved caspases-3, cleaved caspases-8, Bim in NC, D, R and D+R groups by western blot. **(C)** The expressions of EMT-related proteins PD-L1, TRIM21, Vimentin, E-Cadherin, Snail and N-Cadherin in NC, D, R and D+R groups by western blot. The values are expressed as means ± SEM. ^a^*p*<0.05 vs. the NC group, ^b^*p*<0.05 vs. the D group, ^c^*p*<0.05 vs. the R group.

To verify whether DHA has a synergistic antitumor effect in combination with radiotherapy, a Lewis lung cancer mice model was established and treated with DHA at a dose of 50 mg/kg (D group), radiation at 2 Gy delivered once a day for 5 days at 2 Gy of the fractionated radiotherapy on day 1 (R group), DHA and radiation with the same dose of D and R group (D+R group), and no treatment (NC group). As shown in **Figure 6A**, DHA alone had no significant impact on tumor growth, whereas radiotherapy slightly slowed tumor progression. Most notably, integrated DHA and radiation treatment significantly inhibit the tumor growth in comparison with that of DHA alone (*p<0.01*) or radiation alone (*p<0.01*).

**Figure 7:**
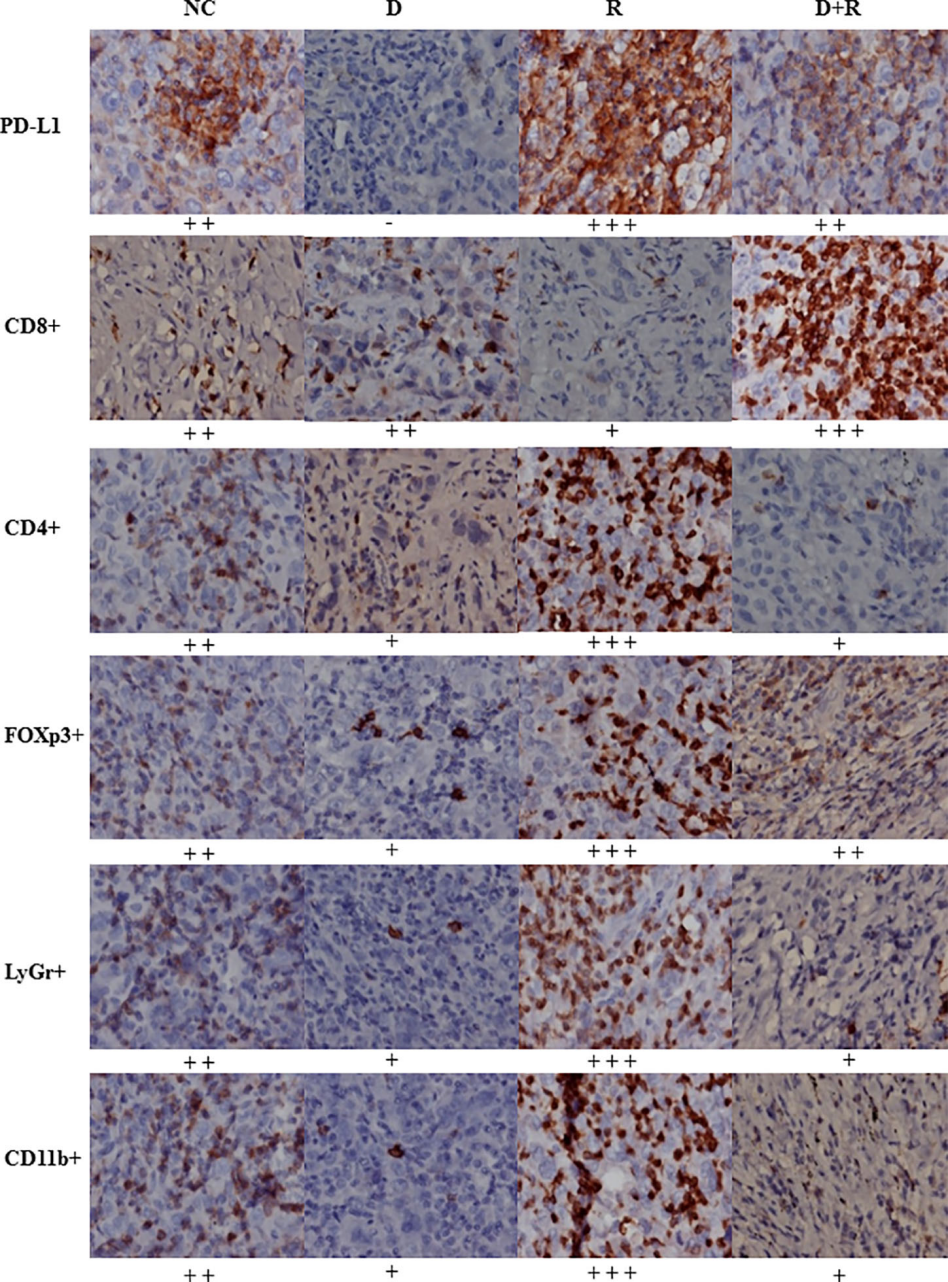
The expressions of PD-L1, CD8+, CD4+, FOXp3+, LyGr+, CD11b+ cells in NC, D, R and D+R groups by immunohistochemistry, scale bar=50 μm.

The western blot results showed that the pro-apoptosis protein BIM was significantly upregulated, anti-apoptosis protein Bcl-2 was downregulated, TRIM21 protein was increased and the cleavage of caspases-8 and caspases-3 was promoted after treatment of radiotherapy and DHA (**Figures 6B**, **C**). The myeloid-derived suppressor cells (MDSCs) have already been proven to be able to suppress immune responses and facilitate tumor progression. The tumor-infiltrating regulatory T cells (iTregs) can suppress the effector T cells and are also the key mediators of peripheral tolerance. In this study, we examined the expressions of PD-L1, CD8+, MDSCs, and iTregs after treatment of DHA and radiation by using immunohistochemistry method. Judging from the immunohistochemistry results, the PD-L1 expression was significantly increased after radiotherapy, but it was inhibited after integrated DHA treatment. By contrast, the expressions of CD8+ cells presented the opposite results, which were enhanced after treatment of integrated DHA and radiation. We also found that these two key mediators of peripheral tolerance, iTregs (CD4+, Fox3p+) and MDSCs (LyGr+, CD11b+), were significantly reduced after treatment of DHA and radiation in mouse model (**Figure 7**). Taken together, integrated DHA and radiotherapy synergistically enhanced antitumor effect through reducing the accumulation of MDSCs and iTregs and promoting CD8+ T cells filtration by downregulating the PD-L1 expression.

## Discussion

Our previous study has already suggested that PD-L1 may be a marker to predict radiation resistance and treatment response to radiotherapy in NSCLC, and combined radiotherapy and anti-PD-L1 antibody could synergistically enhance antitumor effects in NSCLC (19). However, it did not elaborate the specific molecular mechanism of promoting radiation resistance systematically. In this study, a total of 57 patients were enrolled to deliver fractionated radiotherapy, 39 patients with high PD-L1 expressions had radiation resistance, and 18 with negative PD-L1 expression achieved good response to radiotherapy. We also observed that the original PD-L1 negative patients produced radiotherapy resistance after multiple radiotherapies, whose PD-L1 expression in tumor tissue was increased. The NSCLC cell experiments also proved that PD-L1 expressions of non-killed tumor cells in NSCLC were enhanced after radiotherapy and positively related with the radiation resistance.

It already has been indicated that the radiotherapy effects are enhanced in combination with anti-PD-L1 antibody in the NSCLC cells and mice in our previous study (19). However, anti-PD-L1 antibody often induces some unpredicted side effects, and many patients cannot derive survival benefits from high medical expenses (11, 22). It was reported that artemisinin can inhibit tumor growth and enhance the radiation response through triggering production of reactive oxygen species and inhibiting glutathione-S-transferase or inducing a caspases-independent apoptosis-like cell death or causing cell cycle arrest (25, 38). Our previous study demonstrated that artemisinin might be a potential radiosensitizer through regulating the expression of G2 checkpoint-related proteins such as Wee 1 and cyclin B1 in human cervical cancer cells (20). Zhao et al. also revealed that artesunate enhanced radiation sensitivity of A549 cells *via* NO signal transduction pathway to induce cell cycle arrest at the G(2)/M phase (36). DHA is an active metabolite of artemisinin and five times more potent against malaria than artemisinin, which is a safe, effective, FDA-approved and WHO-recommended mainstay in treating malaria. Therefore, we speculated that DHA may be a promising treatment of NSCLC as a radiosensitizer in combination with radiotherapy. Intriguingly, DHA has already been proven to have promising antitumor effects in various cancer types *via* inhibiting TGF-β, AKT, and STAT3 pathways and promoting the cleavage of caspases-8 and caspases-3 and downregulating the expression of anti-apoptosis protein Bcl-2 (30, 31, 37, 39, 40). In this study, we find that DHA eliminates the local aggregation of iTregs and MDSCs and promotes the infiltration of CD8+ T cells in the tumor microenvironment, indicating that DHA can synergistically enhance the antitumor efficacy in combination with radiotherapy. To the best of our knowledge, it is the first to report a small molecule as a radiosensitizer eliminating radiation resistance of NSCLC through abrogating immunity escaping and promoting radiation sensitivity *via* inhibiting PD-L1 expression.

Accumulated evidence also demonstrates that the PD-L1 expression could be enhanced by activation of inflammatory signaling pathways, including TGF-β and PI3K/AKT pathways, as well as transcriptional factors, such as NF-κB and STAT3 (4, 6, 12). The PI3K/AKT pathway is essential for the regulation of growth, proliferation, cell cycle, metastasis, apoptosis, and autophagy, and the STAT3 signaling pathways can play a key role in many cellular processes, such as cell growth and apoptosis (41). In the current study, the expressions of TGF-β, pAKT, pSTAT3, and PD-L1 were all inhibited after treatment of DHA, so DHA downregulates the PD-L1 expression by inhibiting the expressions of TGF-β, pAKT, and pSTAT3. However, it was noticed that the expressions of p-STAT3, p-AKT, and NF-ƘB in A549/X and PC9/X cells did not change after transfection of si-PD-L1-RNA. Therefore, it can be inferred that DHA might abrogate immunity escaping of NSCLC through inhibiting the PD-L1 expression *via* inhibiting its upstream inflammatory signaling pathways, PI3K/AKT, STAT3, and NF-ƘB.

The expression of TRIM21 is closely related to the response to cancer and cell apoptosis (42, 43). In our previous study, we found that TRIM21 could directly bind to PD-L1, and its expression decreased in radiation-resistant cell lines (19). After treatment of DHA, the protein expression of TRIM21 increased significantly. The epithelial-mesenchymal transition (EMT) can facilitate cell apoptosis to promote radiation sensitivity. The EMT-related proteins include E-cadherin, vimentin, snail, and N-cadherin, and their expressions can reflect the rate of cell apoptosis. In this study, the E-cadherin expression was significantly increased in A549/X and PC9/X cells after treatment of DHA, and vimentin, snail, and N-cadherin were all decreased, implicating that DHA may enhance radiation sensitivity *via* regulating the EMT process. Furthermore, the pro-apoptosis protein BIM expression was significantly upregulated, the cleavage of caspases-8 and caspases-3 was promoted and the anti-apoptosis protein Bcl-2 expression was downregulated after treatment of radiotherapy and DHA. DHA may enhance radiation sensitivity to facilitate cell apoptosis through activating PD-L1-independent downstream signaling pathways, TRIM21, and EMT-related proteins.

**Figure 8:**
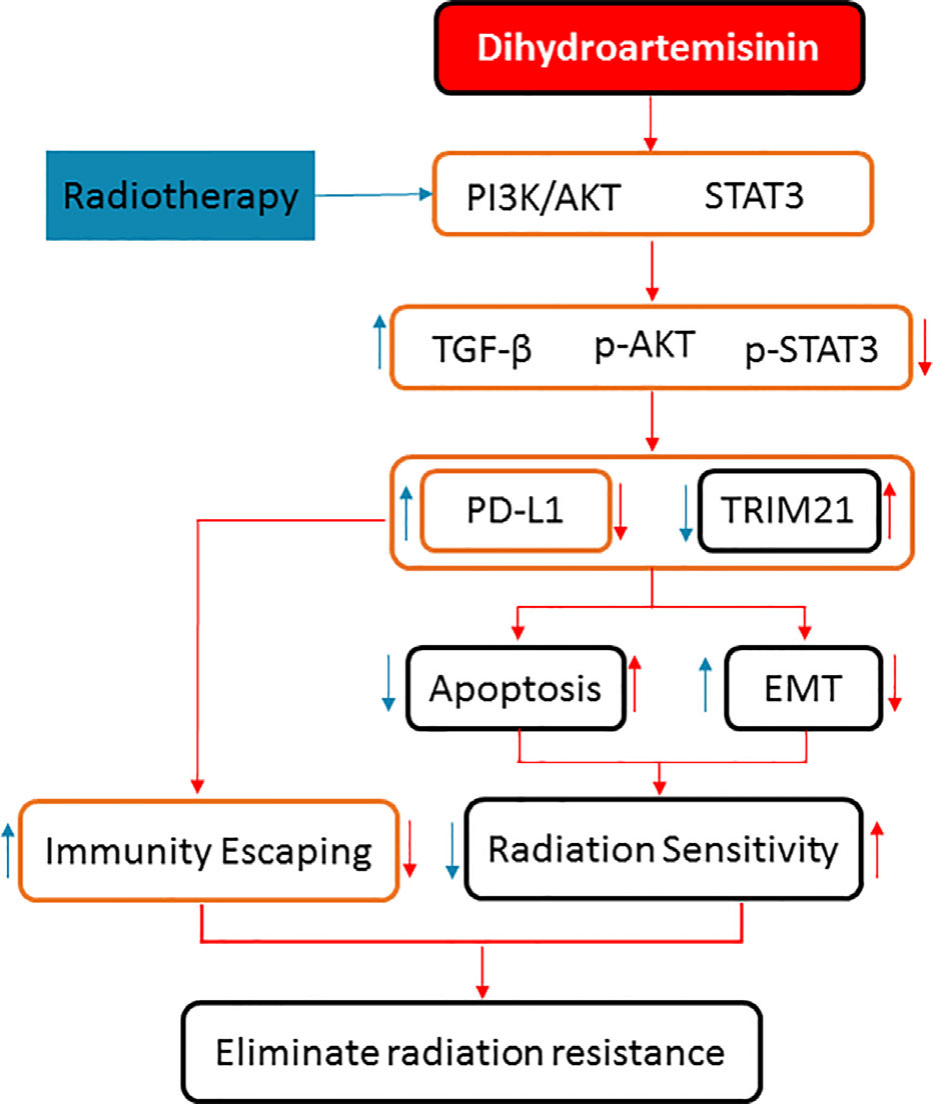
The synergistic mechanisms of DHA enhancing radiotherapy effect of anticancer in NSCLC by inhibiting PD-L1 expression.

To sum up, DHA could eliminate the radiation resistance and synergistically enhance the radiotherapy effect in NSCLC, which was verified in the NSCLC cells and xenograft mice model. The synergistic mechanism of the DHA-enhancing radiotherapy effect is as follows: On the one hand, it could inhibit PD-L1 expression by inhibiting TGF-β, PI3K/Akt, and STAT3 signaling pathways to avoid immune escaping. On the other hand, it could activate by inhibiting the PD-L1-independent signaling pathways, TRIM21, and EMT-related proteins so as to enhance the radiation sensitivity (**Figure 8**). These results provide useful evidence for further exploration of potential clinical application of the combined approach of radiotherapy and DHA. Because DHA is commercially available, and economic, integrated radiotherapy and DHA may provide a promising treatment strategy for the NSCLC patients.

## Data Availability Statement

All datasets presented in this study are included in the article/supplementary material.

## Ethics Statement

The studies involving human participants were reviewed and approved by Ethics Committee of Shanghai Pulmonary Hospital, Tongji University School of Medicine. The patients/participants provided their written informed consent to participate in this study. The animal study was reviewed and approved by Institutional Animal Care and Use Committee of Tongji University School of Medicine.

## Author Contributions

XG contributed to conception and design of the study. HZ, FZ, and HX collected and assembled data in early work, and YW made acquisition of data in the process of perfecting the manuscript. In addition, YW, BS, and YY performed the statistical analysis before submission, and SL and LM made contributions to the analysis of data during revising the manuscript. XG and KW provided study materials and administrative support. YX offered constructive suggestions and supplied the clinical case in improvement of the article. XG wrote the first draft of the manuscript. All authors contributed to manuscript revision, read, and approved the submitted version.

## Funding

This research was supported by the National Natural Science Foundation of China (81602657).

## Conflict of Interest

The authors declare that the research was conducted in the absence of any commercial or financial relationships that could be construed as a potential conflict of interest.

## Abbreviations

NSCLC, non-small cell lung cancer; PD-L1, programmed cell death 1 ligand 1; DHA, dihydroartemisinin; TGF-β, transforming growth factor-β; NF-ƘB, nuclear factor-kappa-B; PI3K: phosphoinositide 3-kinase; FFPE: paraffin-embedded archival; EMT, epithelial-mesenchymal transition; STAT3, signal transducer activator of transcription 3; Erk1/2, extracellular signal-regulated protein kinases 1/2; iTregs, tumor-infiltrating regulatory T cells; MDSCs, myeloid-derived suppressor cells; MDSCs, myeloid-derived suppressor cells; ROS, reactive oxygen species; GST, inhibiting glutathione-S-transferase.
